# Eustress and Malondialdehyde (MDA): Role of Panax Ginseng: Randomized Placebo Controlled Study

**Published:** 2017-07

**Authors:** Hayder M. Al-kuraishy, Ali I. Al-Gareeb

**Affiliations:** Department of Pharmacology, Toxicology and Medicine, College of Medicine, Almustansiriya University, P.O. Box 14132, Baghdad, Iraq.

**Keywords:** *Malondialdehyde (MDA)*, *Eustress*, *Panax Ginseng*

## Abstract

**Objective:** The present study aimed at evaluating the effect of Panax Ginseng on malondialdehyde (MDA) serum levels during eustress on healthy volunteers.

**Method:** In this study, 65 healthy volunteers were recruited from students of a medical school, with the mean age of 22.61±3.63 years. The volunteers were divided into 2 groups: Group A included 35 participants who were treated by Panax Ginseng 500 mg/day, which was regarded as the treated group; group B included 30 participants treated by placebo 500 mg/day, which was regarded as the control group. Baseline data were obtained and then one month after the study, the participants were followed with respect to induction of psychological stress through daily psychomotor performance task and visual working memory accuracy testing. Stress was assessed by malondialdehyde (MDA) serum levels.

**Results:** The participants in the control group showed significant increases in MDA serum levels (p = 0.0004), which were related to significant increases in perceived stress scale from p<0.0001, while Panax Ginseng led to significant reduction in MDA serum levels (p<0.01), with significant increase in perceived stress scale (p = 0.02).

**Conclusion:** Panax Ginseng produced significant reduction in oxidative stress and augmented eustress level in healthy volunteers 1 month after therapy.

Stress is a state in which a person is unable to completely adapt to stressors. Low level of stress may be of value and beneficial in improving physical performance because stress plays a role in motivation and reaction to the environmental stimuli (1). Stress may be externally linked to the environment or internally to the negative stressful emotions (2). Positive psychological stress is called eustress because it leads to considerable motivation, whereas negative psychological stress is called distress because of induction of anxiety and grief (3). Chronic stress leads to induction of oxidative stress (4). Several theories explain the mechanisms of psychological oxidative stress, which may be linked with serotonin and GABA receptors (5). 

Several investigations revealed a relation between oxidative stress and incidence of psychological disorders 

such as schizophrenia, bipolar disorder, and depression due to imbalance between free radicals and antioxidants that cause abnormality in serotonin and GABA receptors (6, 7). 

Depressive perceptions associated with death have been reported across ages, cultures, and religions, making it a global concern for practitioners and medical professionals who interact closely with patients (6).

Humans are recognized to report anxiety and/or despair relating to the reality of death following a life threatening event (7). Cardiac events, in particular, acute myocardial infarction (AMI) are often accompanied by severe psychological sequelae (8).

Augustyniak et al. experimental study revealed that central nervous system is highly susceptible to the oxidative stress due to high brain metabolic and neuronal membrane that contains a large amount of fatty acids oxidized by free radicals. Cerebrospinal fluid contains a large amount of ascorbic acids and iron that are regarded as a source of toxic free radicals (8). 

Psychological stress due to external psychomental stimuli is linked with stimulation of malondialdehyde (MDA) production due to triggering oxidative free radical formations. Mental stress in medical students during examination contributes to the induction of oxidative stress and elevation of MDA serum levels. Thus, overwhelming psychological stimuli activate neuronal oxidative phosphorylation at mitochondrial site, leading to imbalance between pro-oxidant and antioxidant levels, causing profound lipid peroxidation (9, 10). Moreover, cognitive impairment is correlated with high free radical generations and low antioxidant capacity, indicating an association between the neuropathology and oxidative stress (11). Furthermore, oxidative stress markers are malondialdehyde (MDA), metalloenzymes, and selenium dependent glutathione peroxidase (12). However, MDA is regarded as a significant intermediate of hydroxyl radical, causing neuronal dysfunction and degeneration as MDA is a serious neuronal toxin (13). Brain oxidative stress is normally ameliorated and eliminated by free radical scavenger mechanisms including superoxide dismutase and glutathione. Thus, administration of Panax Ginseng or other antioxidants leads to significant activation of antioxidant activity and reduction of MDA serum levels (14).

Therefore, the present study aimed at evaluating the effect of Panax Ginseng on malondialdehyde (MDA) serum levels during eustress on n healthy volunteers.

## Materials and Methods

This study was conducted in Department of Clinical Pharmacology and Therapeutic, College of Medicine, Al-Mustansiriyia University from March to August 2016. The study was permitted and approved by Ethical Committee and Scientific Jury in the College of Medicine, Al-Mustansiriyia University.

***Study Design***

In this single blind study, 65 healthy volunteers were recruited from medical students at the School of Medicine with the mean age of 22.61±3.63 years (29 females and 36 males). The volunteers were divided into 2 groups.

Group A: In this group, 35 participants (20 males and 15 females) were treated by Panax Ginseng 500 mg/day; this group was regarded as the treated group.

Group B: In this group, 30 participants (16 males and 14 females) were treated by placebo (starch capsule) 500 mg/day; this group was regarded as the control group. Baseline data were obtained and 1 month after the study all volunteers in both groups were followed with respect to the induction of psychological stress by daily psychomotor performance task and visual working memory accuracy testing performed by the researchers. Psychomotor performance was estimated by Leeds Psychomotor Battery Tester, and visual working memory accuracy testing was estimated by N-back working memory test. These tests had 90% validity and reliability because of their intra-individual and inter-individual variations (15).

Eustress was evaluated by Perceived Stress Scale (PSS) (16), while stress-induced oxidative stress was assessed by malondialdehyde (MDA) serum levels.

*Psychomotor performance task measures the following variables*:

Total Reaction Time (TRT) is the time needed to react from the start of stimulation to the end of movement action.

Recognition Reaction Time (RRT) is the time needed to recognize the stimuli to the beginning of motor action.

Movement Reaction Time (MRT) is the time needed from beginning of motor action to the end of reaction to the stimuli. All measures were in milliseconds.

Visual working memory accuracy (VWMA) was estimated by computerized visual working memory accuracy, which measures the following variables:

1-Back Model: The volunteer should remember the location of the Blue Square, which was one trail back on the laptop screen.

2-Back Model: The volunteer should remember the location of the Blue Square, which was 2 trails back on the laptop screen.

3-Back Model: The volunteer should remember the location of the Blue Square, which was 3 trails back on the laptop screen. All measures were in percentage.

*Assessment of Perceived Stress Scale (PSS)*

PSS was done by a specific questioner that contained 14 items assessing the stress level of the enrolled healthy volunteers; the scales were 0 = never, 1 = almost never, 2 = sometimes, 3 = fairly often, and 4 = very often. Education level was assessed by a direct interview asking about the number of books read and general knowledge. 

Assessment of Malondialdehyde (MDA) Serum Levels 

**Figure 1:**
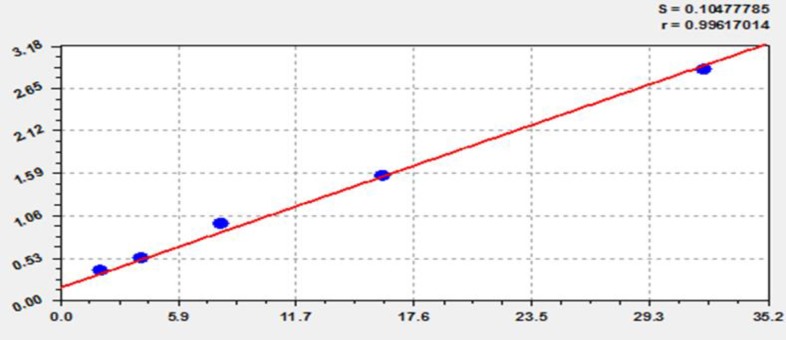
MDA Standard Curve

Blood samples (5ml) were collected from each volunteer via vein puncture technique before and after the treatment to determine malondialdehyde (MDA) serum levels. Blood samples were located in plane tubes at room temperature to clot for 10 minutes, then, separated to get sera via centrifugation at 3000 / rpm for 20 minutes. The sera were stored at (-20◦C), and MDA serum levels (nmol/ml) were estimated by ELISA kit method (Shanghai yehua biological technology Co., Ltd. China). The absorbance was read at 450 nm wavelength to calculate the concentration of standards and corresponding samples (Figure 1).

***Statistical Analysis***

Data were presented as mean ± SD, numbers and percentages. Paired student t test was used to estimate the differences before and after treatment with either placebo or Panax Ginseng. Pearson Correlation coefficient was used to assess the correlation between perceived stress scale (PSS) and malondialdehyde (MDA) serum levels. P value was considered significant when it was less than 0.05. 

## Results

In the present study, all volunteers continued the study without any withdrawal rate,; 55(84.61%) of the enrolled volunteers had a high education level, 6(9.23%) of them had a moderate education level, and 4 (6.15%) of them had low education level, . most Most of the enrolled volunteers were medical students 55(84.61%), while; 10(15.38%) of enrolled volunteers were medical students with extra work additionally; of the participants, 13(20%) of them were smokers, (table Table 1).

Placebo produced insignificant effects on psychomotor performances and visual working memory accuracy p>0.05, . whileHowever,; Panax Ginseng produced significant improvement in the psychomotor performance variables, and it decreases decreased TRT from 648.55±88.22 msc to 512.66±63.19 msc, RRT from 420.68±35.66 msc to 321.95±28.75 msc, and MRT from 227.87±22.99 msc to190.71±18.66 msc p<0.0001. In addition, Panax Ginseng improves improved visual working memory accuracy at 1, 2, and 3-back significantly p<0.0001, as shown in (table Table 2).

Regarding Considering the effect on the oxidative stress and perceived stress scale, placebo showed significant increases in MDA serum levels from 19.33±5.55 to 28.46±11.94 nmol/ml p, p = 0.0004, which was related with to significant increases in perceived stress scale from 14.44±5.63 to 28.49±8.19, p<0.0001. On the other hand, Panax Ginseng led to significant reduction in MDA serum levels from 20.05±8.12 to 12.74±3.28 nmol/ml, with a significant increment in perceived stress scale from 14.41±3.21 to 19.96±4.82, p = 0.02, (table Table 3).

**Figure 2:**
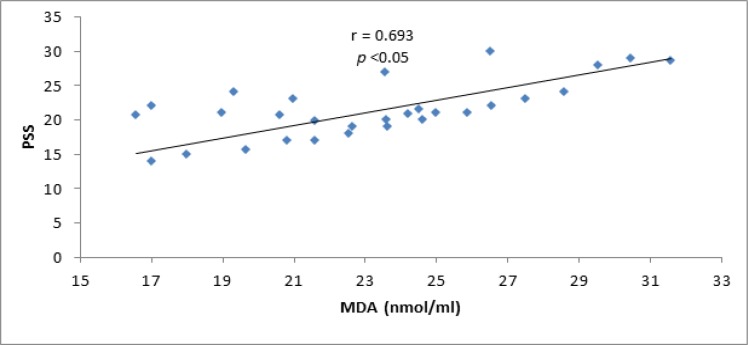
Correlation between MDA serum levels and perceived stress scale

Moreover, perceived stress scale was positively correlated with the oxidative stress as reflected by MDA serum levels (r = 0.693 significant, ly p<0.05 0.05) (figure Figure 2).

**Figure 3:**
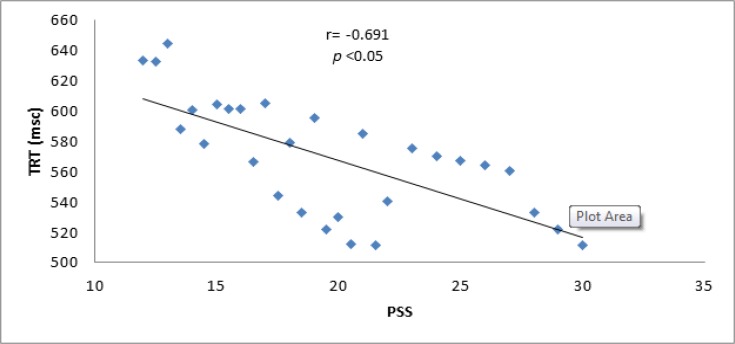
Correlation between TRT and perceived stress scale

Indeed, pPerceived stress scale was negatively correlated with augmented TRT. so;Thus as, as TRT values decreased the perceived stress scale will increased significantly (r = 0.691 p<0.05) (figure Figure 3).

**Table1:** Characteristics of the Study

Variables	Mean±SD/n, %
Number Male: Female ratio Age (years) Race (white: black ratio) High education level Moderate education level Low education level Marital status Single Married Occupation Medical students Medical students with extra work Smokers	65 36:29 22.61±3.63 63:2 55(84.61%) 6(9.23%) 4(6.15%) 63(96.92%) 2(3.07%) 55(84.61%) 10(15.38%) 13(20%)

**Table2:** The Effects of Panax Ginseng on Psychomotor Performances and Visual Working Memory Accuracy Compared to Controls

Variables	Controls (n = 30)		*P*	*Panax Ginseng*(n = 35)			*P*
	Before	After		Before		After	
TRT (ms)	644.90±74.33	642.09±73.12	0.88	648.55±88.22		512.66±63.19	0.0001*
RRT (ms)	422.63±55.11	421.18±53.43	0.917	420.68±35.66		321.95±28.75	<0.0001*
MRT (ms)	222.27±24.05	220.91±27.98	0.840	227.87±22.99		190.71±18.66	<0.0001*
1-back WM (%)	82.34±12.41	83.09±12.73	0.818	83.44±10.55		99.86±15.63	<0.0001*
2-back WM (%)	55.19±10.22	56.22±10.26	0.698	58.44±11.29		70.11±11.78	<0.0001*
3-back WM (%)	30.81±8.89	32.90±8.11	0.345	32.44±7.82	55.39±9.81		<0.0001*

Results are expressed as mean ± SD;

* p<0.01; TRT: total reaction time, RRT: recognition reaction time, MRT: movement reaction time, 1-back WM: one back working memory, 2-back WM: two back working memory, 3-back WM: three back working memory.

**Table 3:** The Effects of Panax Ginseng on MDA Serum Levels and Perceived Stress Sale Compared to Controls

**Variables**	**Controls (n = 30)**		***P***	***Panax Ginseng*** **(n = 35)**		***P***
	**Before**	**After**		**Before**	**After**	
MDA (nmo/ml)	19.33±5.55	28.46±11.94	0.0004	20.05±8.12	12.74±3.28	<0.0001*
PSS	14.44±5.63	28.49±8.19	<0.0001*	14.41±3.21	19.96±4.82	0.02"

Results are expressed as mean ± SD;

" *p*<0.05

* *p*<0.01; MDA: malondialdehyde, PSS: perceived stress scale

## Discussion

The present study demonstrated that frequent daily exposure to the positive and motivating psychological stressors leads to augmentation of perceived stress score and oxidative stress mediator (MDA) levels as oxidative stress like reactive oxygen species is not only linked with disease initiation and propagation, but it is also regarded as an important signaling messenger. Thus, removal of these oxidative mediators may be detrimental to the human health (17). 

Oxidative stress naturally occurs during normal biological process that produces harmful reactive species, which attack the cellular membrane and constituents (18). Thus, mental and psychological stresses increase the risk of cognitive dysfunction due to reduction of natural antioxidant system. In addition, improvement in the psychomotor performance ability was related to decline in the oxidative stress and augmentation of endogenous antioxidant ability (19). Padurariu et al. study demonstrated that MDA is a potential marker of lipid peroxidation during cognitive stress and that high MDA serum level is associated with oxidative damage that induced cognitive impairment (20).

Panax Ginseng improves psychomotor performances and visual working memory with significant reduction in MDA serum levels and significant effect on perceived stress scale; these findings are supported by many studies that illustrated the potential role of Panax Ginseng in the treatment of cognitive and memory deficit (21), antioxidant effect (22), and anti-stress effect (23). Moreover,, the present study, showed that placebo produced insignificant effect on the psychomotor performances and visual working memory but with significant increments in MDA serum levels and perceived stress scale since placebo had insignificant antioxidant and anti-stress effects (24). This finding is supported by Asqaryet al. study that illustrated insignificant effect of placebo on oxidative stress and MDA serum levels in the evaluation of the effects of Hibiscus sabdariffa on oxidative stress in patients with metabolic syndrome (25). Additionally, this finding is sustained by Zolfaghari et al. research on unripe grape effect on the oxidative stress and blood pressure in rats that disclosed insignificant effect of placebo on the oxidative stress markers (26). In the present study, Panax Ginseng produced a significant effect on the reduction of MDA serum levels following oxidative stress induced by cognitive and psychological stresses by the ability of Panax Ginseng in the reduction of MDA serum levels. These findings are in agreement with various studies that revealed a potential effect of Panax Ginseng in reduction of MDA serum levels and stimulation of endogenous antioxidant enzyme given that Panax Ginseng improves cellular glutathione peroxidase and nitric oxide activity. Moreover, Panax Ginseng restrained ginsenosides and saponins that possess potent antioxidant activities via scavenging free radicals and inhibition of lipid peroxidation during acute psychological stress (27, 28).

Moreover, Seo et al. study showed that compound K, isolated from Ginseng, possesses significant antioxidant effect, attenuate mitochondrial damage and cytotoxicity induced by glutamate during excitotoxicity induced by neuronal stimulation; it reverses scopolamine induced memory deficit through induction of Nrf2-mediated activity. Thus, Panax Ginseng therapy prevents neurological dysfunction caused by free radicals (29). Panaxynol derived from Ginseng is regarded as a potent activator of Nrf2 signaling, regulates cytokine expression, and has antioxidant activity (30). These findings may explain the effect of Panax Ginseng therapy in improving psychomotor performance, visual working memory accuracy, reduction of MDA serum levels, and augmentation of positive psychological stress (eustress), compared with placebo. They also signify the importance of eustress and rising in MDA serum levels without improvement in psychomotor performance and visual working memory accuracy; this indicates a positive correlation between MDA and stress scale as disclosed in the present study.

Perceived stress scale was positively correlated with MDA serum levels as psychological eustress is associated with intensification of oxidative stress (31). However, total reaction time was negatively correlated with perceived stress scale, as augmented TRT was associated with high-perceived stress scale (32). Therefore, eustress or positive psychological stress was able to induce oxidative stress attenuated by Panax Ginseng therapy. Panax Ginseng in our study produced a mild significant effect on stress scale, which might have been due to the small sample size or significant antioxidant effect which decreased MDA induced eustress and adaptive response as MDA plays a role in autoregulation of cerebral blood flow during brain stimulation (33).

## Limitations

First, this study had a single-blind technique, which might have biased the results. Second, gender differences were not estimated. Third, other stress scales were not estimated, so we could not make any comparisons among the scales. Fourth, only young participants were included in this study. All these factors might have limited our results. . 

## Conclusion

Panax Ginseng produced a significant reduction in oxidative stress and augmented eustress level in normal healthy volunteers one month after therapy
